# The development of early social cognitive skills in neurogenetic syndromes associated with autism: Cornelia de Lange, fragile X and Rubinstein–Taybi syndromes

**DOI:** 10.1186/s13023-021-02117-4

**Published:** 2021-11-22

**Authors:** Katherine Ellis, Jo Moss, Chrysi Stefanidou, Chris Oliver, Ian Apperly

**Affiliations:** 1 School of Psychology, University of Birmingham, Birmingham, B15 2TT UK; 2 School of Psychology, University of Surrey, Guildford, Surrey, GU26 7XH UK; 3 Faculty of Health, Education, Medicine and Social Care, Anglia Ruskin University, Rivermead Campus, Bishop Hall Lane, Chelmsford, CM1 1SQ UK

**Keywords:** Development, Intellectual disability, Rare systemic diseases, Autism, Cornelia de Lange syndrome, Fragile X syndrome, Rubinstein–Taybi syndrome, Social cognition

## Abstract

**Background:**

Cornelia de Lange (CdLS), Fragile X (FXS) and Rubinstein–Taybi syndromes (RTS) evidence unique profiles of autistic characteristics. To delineate these profiles further, the development of early social cognitive abilities in children with CdLS, FXS and RTS was compared to that observed in typically developing (TD) and autistic (AUT) children.

**Methods:**

Children with CdLS (*N* = 22), FXS (*N* = 19) and RTS (*N* = 18), completed the Early Social Cognition Scale (ESCogS). Extant data from AUT (*N* = 19) and TD (*N* = 86) children were used for comparison.

**Results:**

Similar to AUT children, children with CdLS, FXS and RTS showed an overall delay in passing ESCogS tasks. Children with CdLS showed a similar degree of delay to AUT children and greater delay than children with FXS and RTS. The CdLS, FXS and RTS groups did not pass tasks in the same sequence observed in TD and AUT children. Children with CdLS (*p* = 0.04), FXS (*p* = 0.02) and RTS (*p* = 0.04) performed better on tasks requiring understanding simple intentions in others significantly more than tasks requiring joint attention skills.

**Conclusions:**

An underlying mechanism other than general cognitive delay may be disrupting early social cognitive development in children with CdLS, FXS and RTS. Factors that may disrupt early social cognitive development within these syndromes are discussed.

**Supplementary Information:**

The online version contains supplementary material available at 10.1186/s13023-021-02117-4.

## Background

Social cognition encompasses a range of cognitive abilities that enable understanding the intentions, thoughts, beliefs and behaviours of others that are fundamental to successful social interaction [1]. Autism spectrum disorder is diagnosed based on the presence of both “persistent difficulties with social communication and social interaction” and “restricted and repetitive patterns of behaviours, activities or interests” [2]. Throughout the manuscript we use the term ‘autism spectrum condition’ (ASC) to avoid medicalised language as preferred by the autistic community [3]. Social communication and interaction difficulties observed in autistic individuals are, arguably, driven by atypical social cognition also observed in these individuals [4]. Given this association, it is possible that compromised social cognition might underpin atypical social behaviours and other related characteristics, such as social anxiety, described in a number of neurogenetic syndromes associated with intellectual disability and ASC [5–9].

Cornelia de Lange (CdLS), fragile X (FXS) and Rubinstein–Taybi (RTS) syndromes are rare neurodevelopmental conditions of genetic cause. CdLS is caused by genetic variants of the *NipBl*, *SMC1A*, *SMC3*, *RAD21*, *BRD4*, *HDAC8* and *ANKRD11* genes and it’s estimated to occur between 1 in 10,000 and 1 in 30,000 live births [10]. Full mutation FXS is caused by a sequence of 200 or more cytosine-guanine-guanine (CGG) trinucleotide repetitions on chromosome Xq27.3. More men (1:4000) than women (1:6000) have fragile X syndrome because FXS is an X-linked syndrome leading to more males being affected than females [11]. RTS is caused by de novo deletions [12] or heterozygous mutations [13] on *CREBBP* and *EP300* genes on chromosome 16p.13.3 and affects between 1:100,000 and 720,000 newborns [14].

Whilst individuals with CdLS, FXS and RTS have a heightened likelihood of showing autistic traits compared to the general population [15, 16], these groups show distinct profiles of these traits [17]. Individuals with CdLS show fewer repetitive behaviours but more frequent and greater communication difficulties than individuals with non-syndromic ASC [17], whereas individuals with FXS show an even profile of ASC-related social interaction, communication and repetitive behaviours that is a similar pattern but at lower levels compared to autistic individuals [18, 19]. In contrast, the profile of autistic characteristics in individuals with RTS is defined more by restricted and repetitive behaviours than social and communication impairments [16]. These differences may be indicative of variability across aspects of social cognition that underpin the profiles of social behaviour and communication that are associated with ASC in these syndromes.

To date, investigation of social cognition in individuals with CdLS and FXS has demonstrated delayed performance in passing traditional single task paradigms evaluating false belief understanding i.e. the understanding that an agent may hold a belief that is both different from the participants and contradictory from reality [20], relative to mental age [21–24]. However, a single cognitive paradigm is unable to account for the detailed differences in profiles of social interaction skills and behaviours observed across these syndromes. For example, boys with FXS show similar performance on false belief tasks to boys with Down syndrome [22], a syndrome characterised by high levels of sociability [25] but with more subtle difficulties in social interaction and developing friendships [26].

A range of social cognitive abilities, such as shared intentionality and explicit mentalising abilities, develops from infancy [27] to adolescence [28]. The development of these abilities in typically developing (TD) children conforms to a strict developmental sequence evidenced by scaling analysis [27, 28]. These developmental scales provide a robust normative benchmark to compare the development of social cognitive abilities in groups associated with differences in profiles of social interaction skills and behaviours across development [27, 29]. Importantly, they can determine whether specific abilities: (1) are delayed relative to mental age, and/or (2) emerge in a different sequence from that observed in TD children.

Both delay and differences in social cognitive development have implications for refining hypotheses regarding the underlying mechanisms that drive the development of abilities in atypical groups [30]. For example, both late-signing deaf children and AUT children demonstrate delayed scale progression on the *Theory-of-Mind Scale* (ToMS) but only the AUT children passed tasks in a divergent order from TD [31, 32]. The authors hypothesised that whereas similarities in overall delay may reflect broad similarities in atypical social experiences between AUT children and late-signing deaf children that do not offer as many opportunities to learn about other’s mental states, the divergent developmental sequence in AUT children may reflect group specific neurobiological or environmental (e.g. teasing) influences that make processing hidden emotion easier or more relevant to their day-to-day life than other’s false beliefs.

A large body of literature indicates that some of the earliest developing social cognitive abilities enable infants to form shared intentionality with others to cooperate and coordinate their interactions and achieve joint goals [27, 33]. The Early Social Cognition Scale (ESCogS, [27]) is a developmental scale that assesses behaviours with different kinds of underlying intentional structure that typically emerge in TD children between the ages of 14 to over 24 months. Thus, the ESCogS is currently the only scale that enables assessment of social cognitive abilities even in children who are very cognitively delayed, including those with CdLS, FXS and RTS. Abilities range from the understanding of basic goal directed actions to more sophisticated cooperative and joint problem-solving abilities that require ‘shared intentionality’ and the formation of joint goals with others. Application of the ESCogS has demonstrated that whilst autistic children with a developmental delay passed early social cognitive abilities in the same sequence as TD infants, they showed a delay in performance relative to their overall ability specifically on tasks requiring them to follow others’ eye gaze and to cooperate with others [27]. This demonstration provides proof of concept that the ESCogS enables the investigation of social cognitive abilities that may underpin profiles of social interaction skills and behaviours. We further explore this concept by applying this tool to investigate whether there are different profiles of delay or difference in early social cognitive development across neurogenetic syndromes associated with distinct profiles of ASC-related social and communication skills.

The aim of the current study is to examine the development of early social cognitive abilities in children with CdLS, FXS and RTS, syndromes associated with different profiles of autistic characteristics. We compared these groups to (1) TD infants to determine whether social cognitive development in these groups is atypical and (2) a group of autistic (AUT) children to explore similarities or differences in social cognitive development. The developmental trajectory of early social cognitive skills in individuals with CdLS, FXS and RTS relative to participants’ non-verbal mental age will be determined, to establish whether these skills develop at a rate that is advanced, similar or delayed to TD infants and autistic children. Additionally, we will determine whether CdLS, FXS and RTS develop social cognitive abilities in the same order as that observed in TD and autistic children and/or one another or in a different order.

It was hypothesised that:

1. As individuals with CdLS and FXS show delay in passing false belief tasks [21–24], these groups will show a delay in acquiring earlier developing abilities in the present study. As social cognition has not been investigated in individuals with RTS, investigation is exploratory.
2. Due to their unique profiles of autistic traits [17, 18], individuals with CdLS, FXS and RTS will not develop these skills in the same order as that observed in TD and AUT children.

## Method

### Recruitment

Participants were recruited as part of a wider study investigating social cognition and social behaviour in neurogenetic syndromes and follow the same procedures outlined in Ellis et al. [29]. Participants were contacted via an existing database and the study was advertised via syndrome support groups. Participants were included if they had received a clinical diagnosis of their syndrome by a paediatrician or a clinical geneticist and they were aged under 16 years. As per the requirements of the Autism Diagnostic Observation Schedule 2nd edition [34], we included participants older than 30 months with communication and motor age equivalence of 15 months or above on the Vineland Adaptive Behavior Scales Second Edition (Vineland-II; [35]). Participants younger than 30 months had a non-verbal mental age of at least 12 months. Only males were included in the FXS group due to sex differences in social behaviour [36, 37].

### Participants

Participants were twenty-two children with CdLS (M_age_ = 77.98 months, SD = 39.46), nineteen with FXS (M_age_ = 71.70 months, SD = 30.40) and eighteen with RTS (M_age_ = 110.61 months, SD = 45.95) aged between 2 and 15 years. Parents and legal guardians provided informed written and verbal consent on behalf of their child. Children who had capacity gave verbal consent. Ethical approval for this study was granted by the Science, Technology, Engineering and Mathematics Ethical Review Committee at the University of Birmingham (approval number: ERN_12-0017AP16).

Extant data from twenty AUT children (M_age_ = 104.18 months, SD = 35.76) and eighty-six typically developmental children (M_age_ = 22.03 months, SD = 5.32) were used to provide comparative data for some analyses. Some of these participant’s data are reported in Ellis et al. [27]. One AUT participant was removed from the original sample as their non-verbal mental age was substantially higher than other participants in the AUT group and another was removed because their non-verbal mental age was not available.

Table 1 shows that whilst the groups were not comparable on chronological age; the syndrome and AUT groups were significantly older than the TD infants and the FXS group was significantly younger than the AUT and RTS groups, there were no significant group differences on non-verbal mental age between the AUT, CdLS, FXS and RTS groups. All these groups had a significantly higher mental age than the TD group. Syndrome groups were comparable on the Autism Diagnostic Observation Schedule Second Edition (ADOS-2; [34]) calibrated severity scores but not comparable on sex as we did not include girls with FXS. The AUT group also had fewer girls than the CdLS and RTS groups, although these proportions correspond to previous reports of gender ratios of ASC diagnosis [38]. Syndrome groups also differed on adaptive behaviour age equivalents as the CdLS group had a significantly lower age equivalent than the FXS and RTS groups. No significant differences were found for primary carer’s education level or total family income between groups. Information regarding autistic characteristics, adaptive behaviour, as well as family income and primary caregiver’s level of education was not available were not available for the TD and AUT groups. We did not collect information on participant’s race/ethnicity for any of the groups.

**Table 1:** Participant characteristics

	TD (*n* = 86)	AUT (*n* = 20)	CdLS (*n* = 22)	FXS (*n* = 19)	RTS (*n* = 18)	*p*	Post-hoc tests (*p* < 0.05)
Mean chronological age in months (SD)	22.03 (5.32)	104.18 (35.76)	77.98 (39.46)	71.70 (30.40)	110.61 (45.95)	** < 0.01**	TD < FXS, < AUT, RTS, TD < CdLS
Sex % female	47	30	59	0	50	** < 0.01**	FXS < AUT < TD, CdLS, RTS
Mean non-verbal mental age in months (SD)^a^	22.03 (5.32)	35.47 (18.77)	29.62* (13.67)	33.01 (10.42)	30.32** (9.52)	** < 0.01**	TD < AUT, CdLS, FXS, RTS
Mean non-verbal developmental quotient (SD)^a^	NA	38.89 (27.90)	43.85* (16.45)	49.32 (13.95)	34.47** (21.51)	**0.03**	ASD, RTS < FXS
Mean adaptive behaviour age equivalent in months (SD)^b^	NA	NA	22.18 (13.06)	30.55 (13.64)	33.74 (15.53)	**0.03**	CdLS < FXS, RTS
Mean ADOS-2 Calibrated Severity Scores (SD)	NA	NA	4.38 (3.19)***	6.17 (1.50)	4.88 (1.86)	0.08	
Mean Primary carer’s education level band (SD)^c^	NA	NA	4.15** (1.38)	4.17*** (1.20)	4.06 ** (1.34)	0.99	
Mean total annual family income band (SD)^d^	NA	NA	3.65** (1.93)	4.18** (1.59)	3.73* (2.05)	0.48	

Significant *p* values indicating differences between groups are highlighted in bold

Information not available for the relevant measure for: * three participants, ** two participants, *** one participant

^a^Assessed using either the Mullen Scales of Early Learning [39] or the British Ability Scales 3rd edition [40]. Please see below for further information on how cognitive ability was assessed. TD children’s non-verbal mental age was inferred based on their chronological age

^b^Derived by averaging the age equivalents across all Vineland-II subscales appropriate across the age range of participants i.e. receptive and expressive (communication domain), personal (daily living skills domain), and interpersonal relationships, play and leisure, and coping skill subscales (socialisation doman)[35]

^c^Parents indicated the band that best represented their level of educational qualifications of the primary caregiver. Education bands included (1) No formal education qualifications, (2) Fewer than 5 GCSE’s or O’Levels (grades A–C), NVQ1 or BTEC First Diploma, (3) 5 or more GCSEs or O Level’s (Grades A–C), NVQ2 or equivalent, (4) 3 or more ‘A’ Levels, NVQ3, BTEC National, or equivalent, (5) Polytechnic/University degree, NVG4, or equivalent, (6) Masters/Doctoral degree, NVQ5, or equivalent

^d^Parents indicated the band that best represented current total annual family income. Income bands included 1. Less than £15,000, 2. £15,001 to £25,000, 2. £25,001 to £35,000, 3. £35,001 to £45,000, 4. £45,001 to £55,000, 5. £55,001 to £65,000, 7. £65,001 or more

### Measures

Caregivers completed a demographic questionnaire including information on participant’s age, sex, diagnosis, family income and primary caregiver’s level of education. The ADOS-2 was used to assess level of autistic characteristics.

Cognitive ability was assessed with the Mullen Scales of Early Learning [39] (suitable from birth to 5 years, 8 months) and the British Ability Scales Third Edition (BAS3) [40] (3 years to 17 years, 11 months). The BAS3 consists of two batteries: (1) Early Years BAS3 (3 years to eight years, 11 months) and (2) School Age BAS3 (6 years to 17 years, 11 months). Cognitive assessment was chosen based on an individual’s age equivalent scores on the communication and motor domains of the Vineland-II completed by parents prior to the testing session and clinical judgement. If a participant achieved floor or ceiling on a subscale in a domain, and if time and participant’s attention permitted, they took part in the assessment that was appropriate for less/more able individuals respectively for the domain. As many participants could not take part in the cognitive assessment appropriate for their chronological age due to intellectual disability, mental age scores were used to compare overall ability between syndrome groups. Many of those who participated in the BAS3 performed at both floor and ceiling effects on the School Age and Early Years expressive language subscales respectively. Therefore, we used participant’s non-verbal mental age calculated from the mean of participant’s age equivalents on the two non-verbal subscales of the cognitive assessment as a proxy for cognitive ability.

The Early Social Cognition Scale (ESCogS [27] was administered by two experimenters. Tasks ranged from assessments of understanding of basic goal directed actions (Helping), up to assessments of ‘shared intentionality’ and cooperation with another person (Cooperation tasks). Participants are coded ‘pass’ or ‘fail’ for each task. Three tasks (Helping and both Communication tasks) include control trials that are analysed separately from experimental trials to check participants produced target behaviours following interpretations of an experimenter’s intention rather than reinstating the original situation (Helping) or due to low level attentional cues (Communication). Participants completed the tasks in one of six counterbalanced orders (see Additional file 1). Inter-rater reliability between two raters was calculated for 46% of the sample. The mean level of agreement across tasks was 0.9 (ranging 0.71–1.0), indicating very good reliability. See Additional file 2 for summary of tasks and corresponding control conditions and Ellis et al. [27] for a full description of each task.

The Autism Diagnostic Observation Schedule, 2nd edition (ADOS-2) [34] was administered and scored by a research reliable trained examiner to assess ASC characteristics. Calibrated severity scores (CSS) were calculated for each participant, providing a comparison of severity of ASC characteristics relative to a sample of autistic individuals and the same chronological age as the participant. CSS range from scores of one (indicating a low level of ASD symptomatology) to 10 (indicating a high level of symptomatology).

**Fig. 1:**
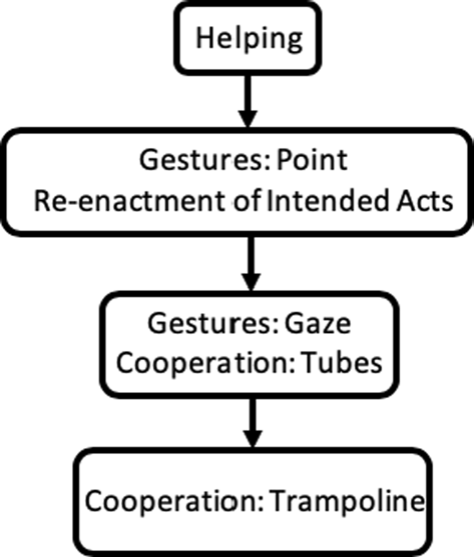
Early Social Cognition Scale [27]

The Vineland Adaptive Behavior Scales-II (Vineland-II, Survey Form) [35] is a semi-structured interview conducted with caregivers, which assesses each participant’s adaptive abilities in four main domains: communication, daily living skills, socialisation and motor skills. Age equivalent scores on the communication and motor scales contributed to the decision of which cognitive assessment was appropriate for that participant to take part in (Fig. 1).

### Procedure

Individuals were assessed either at the University, at their home and/or at syndrome family support group conferences. Caregivers completed the demographic questionnaire and completed the Vineland-II via telephone one week before a visit. Typically, the cognitive assessment was administered first, followed by the ESCogS and finally the ADOS-2. Those who took part at conferences participated in the social cognitive scales first and completed the remaining assessments during a visit.

## Results

### Control trials

Only eight out of the forty-six children who passed the experimental condition also handed the target item to the experimenter during the control condition and only five participants took possession of target items during the experimental trials before handing them over. This suggests that these children helped the adult as opposed to reinstating the original situation or taking the object primarily for themselves [27].

Mann–Whitney U tests revealed that the choices of participants who passed the *Gestures* tasks between containers did not significantly differ from chance in either the Communication: Point (U = 1339.50, *p* = 0.08) or Communication: Gaze trials (U = 1548.50, *p* = 0.64) control trials. These results suggest that children who passed each *gestures* task only followed intentional cues [27].

### Performance on *ESCogS* relative to non-verbal mental age

**Fig. 2:**
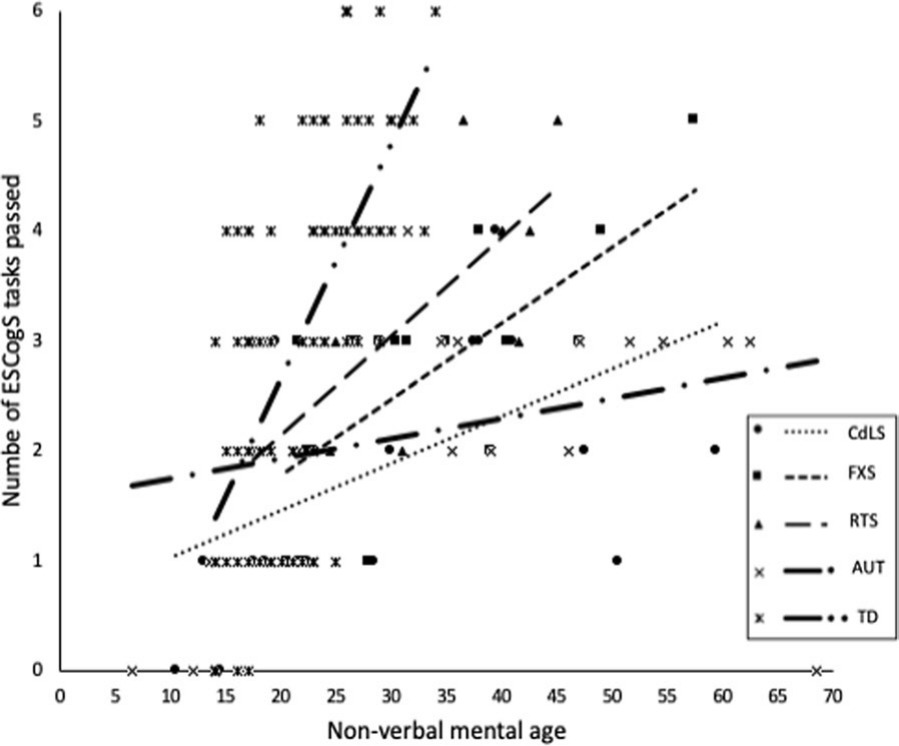
Number of ESCogS tasks each participant passed plotted against their non-verbal mental age

Table 2 shows the number of children who passed each task within each syndrome. To explore whether social cognitive ability was delayed or preserved in CdLS, FXS and RTS, non-verbal mental age (where data were available; see Table 1) was plotted against the number of ESCogS tasks passed for each group and the AUT comparison group. For each TD infant, chronological age was plotted against the number of tasks passed. A line of best fit was included for each group (Fig. 2). Visual inspection of Fig. 2 reveals that all clinical groups show an overall delay in acquiring social cognitive abilities in comparison to the chronological age at which TD infants pass these tasks. However, individuals with FXS and RTS showed greater development of early social cognitive ability compared to AUT children and children with CdLS.

**Table 2:** Frequency of children per group that passed each task ordered by difficulty for TD children

	TD (*N* = 86)*	AUT (*N* = 21)*	CdLS (*N* = 22)	FXS (N = 19)	RTS (N = 18)
Helping	76 (88%)	16 (76%)	14 (64%)	16 (84%)	16 (89%)
Communication: point	58 (67%)	13 (62%)	6 (27%)	6 (32%)	8 (44%)
Re-enactment of intended acts	54 (63%)	13 (62%)	14 (64%)	16 (84%)	15 (83%)
Communication: Gaze	37 (43%)	4 (19%)	2 (9%)	3 (16%)	1 (6%)
Cooperation: Tubes	32 (37%)	2 (10%)	3 (14%)	7 (37%)	6 (33%)
Cooperation: Trampoline	19 (22%)	0 (0%)	2 (9%)	3 (16%)	7 (39%)

*Percentages for the TD and AUT group as reported in Ellis et al. [27]

Correlations were run to determine whether, despite delay, overall social cognitive ability increased with non-verbal mental age and chronological age. Kendall Tau correlations revealed moderate positive correlations in the CdLS (τ_b_ = 0.45, *p* = 0.01), FXS group (τ_b_ = 0.50, *p* < 0.01) and RTS groups (*r* = 0.68, *p* < 0.01) indicating that higher non-verbal mental age was associated with more social cognitive tasks passed. No significant correlation between non-verbal mental age and number of tasks passed in the AUT group was found. Whereas a moderate positive correlation was found between age and number of tasks passed in the TD groups (τ_b_ = 0.53, *p* < 0.01), no significant correlations were found for the AUT, CdLS, FXS and RTS groups. Findings indicate that participant’s non-verbal age, but not chronological age is associated with the rate of social cognitive development in these clinical groups.

A Kruskal–Wallis test indicated a significant group difference between groups for the number of tasks passed (χ(4) = 15.23, *p* < 0.01). Post-hoc Mann Whitney U tests revealed that TD infants (Mean = 3.10, SD = 1.70) passed significantly more tasks than children with CdLS (M = 1.86, SD = 1.21) (U = 539.00, z = − 3.12, *p* < 0.01, r = − 0.30) and AUT children (M = 2.15, SD = 1.18) (U = 558.50, z = − 2.47, *p* = 0.01, r = − 0.24). Individuals with CdLS passed significantly less tasks than individuals with FXS (M = 2.68, SD = 1.06) (U = 131.50, z = − 2.11, *p* = 0.04, r = − 0.33) and RTS (M = 2.89, SD = 1.28) (U = 113.50, z = − 2.37, *p* = 0.02, r = − 0.36). No differences were found between the CdLS and AUT groups, the RTS and AUT groups, the TD and FXS groups or the TD and RTS groups. Additionally, no differences were found between the FXS and RTS group despite differences in chronological age (Table 1).

Findings suggest that having a higher non-verbal mental age than the chronological age of the TD infants did not mean that the AUT, FXS and RTS groups passed more tasks relative to TD infants. AUT children and children with CdLS showed a significant delay relative to TD infants.

### Guttman scaling analyses

Guttman scaling analysis was conducted to explore whether those within each syndrome group developed social cognitive abilities in the same order as that observed in TD infants and AUT children [27]. Scaling establishes whether a specific sequence (i.e., one in which children will pass all tasks in order of difficulty up to a certain task dependent on their developmental stage and subsequently fail any task that is more difficult past that point) emerges reliably *within* children. It has advantages for examining the abilities of syndrome groups because scaling provides a criterion for assessing the typicality of a group’s performance without any need for matching or quantitative comparison between groups [28].

Tasks that are both attained at a similar age i.e., Re-enactment of Intended Acts and Communication: Point [41, 42], and Communication: Gaze and Cooperation: Tubes tasks [42, 43] were placed on a step of equal difficulty. Children were coded as having passed that step if they had passed either of the tasks of equal difficulty [27].

The co-efficient of reproducibility (Rep) for each group indicates how much the sequence of passes and fails fit into a perfect Guttman scale by measuring how many responses deviate from this ideal scale. As it is unlikely to attain a perfect scale across all participants, an approximation of the perfect scale is 0.9 or above—i.e. the data are at least 90% reproducible [51, 52]. The index of consistency (IoC) estimates whether the observed co-efficient of reproducibility is significantly greater than that achieved by chance. An IoC of 0.5 or more is considered scalable.

Table 3 shows the scalogram sequences previously observed in infants and AUT children, and the percentage of children in each syndrome group whose responses fitted each sequence perfectly. Although many individuals fitted one of the expected sequences (73% of children with CdLS, 74% with FXS and 78% with RTS), results indicate that none of the syndrome groups passed the tasks in the same scalable fashion as TD infants. Whilst the Rep was > 0.90 for all groups (CdLS = 0.93, FXS = 0.93, RTS = 0.94), the IoC did not reach the 0.5 criteria in any group (CdLS = 0.32, FXS = 0.11, RTS = 0.41). This is supported by visual inspection of the pass and fails for each step (see Additional file 3), which reveals errors in all syndrome groups where participants fail supposedly easier tasks despite passing supposedly more difficult ones.

**Table 3:** Frequency of children with CdLS, FXS and RTS who fit the original Guttman sequence

Sequence	0	1	2	3	4	Other patterns	*N*	*N* fit scale exactly
Helping	–	+	+	+	+			
Re-enactment of intended acts or Communication: Point	–	–	+	+	+			
Communication: Gaze OR Cooperation: Tubes	–	–	–	+	+			
Cooperation: Trampoline	–	–	–	–	+			
Syndrome								
CdLS	2	3	7	2	2	6	22	16 (73%)
FXS	0	0	7	5	2	5	19	14 (74%)
RTS	0	2	5	2	5	4	18	14 (78%)
AUT*	3	1	13	2	0	2	21	19 (90%)
TD*	4	7	19	29	14	13	86	73 (85%)

A plus sign indicates that a child passed a task, whereas a minus sign indicates that a child failed a task

*As reported in Ellis et al. [27]

### Exploring alternative developmental sequences

**Fig. 3:**
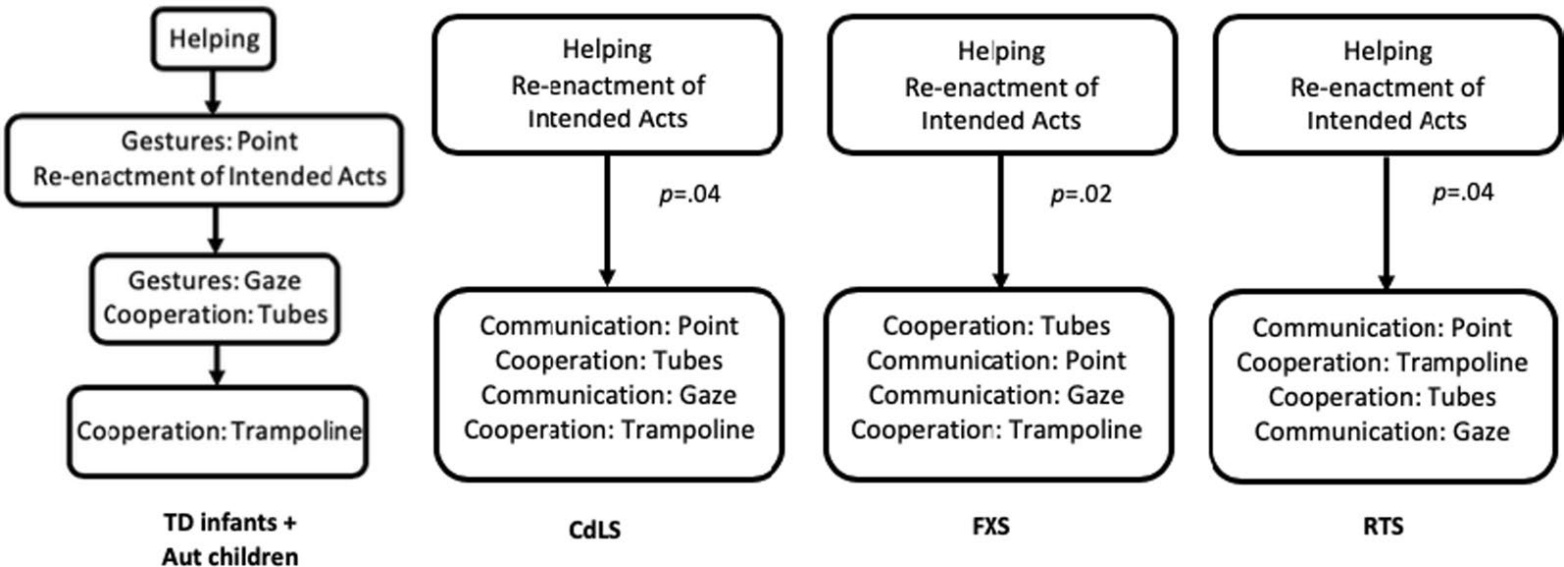
Sequence that children with CdLS, FXS and RTS passed ESCogS tasks

The scalogram analysis provided no evidence that individuals with CdLS, FXS, and RTS showed the same pattern of relative ease and difficulty between tasks as TD individuals, but it casts no light on why this might be the case. Inspection of the frequencies of correct responses suggested that, while in TD participants Communication: Point grouped with Re-enactment of Intended Acts in the second step of the scale, in individuals with CdLS, FXS, and RTS the Communication: Point task appeared more difficult. To test this and whether individuals with CdLS, FXS and RTS develop social cognitive abilities in a reliable but alternative progression to TD infants and autistic children, tasks were ordered in increasing difficulty for each syndrome group and differences between performances on each consecutive pair of tasks were tested with McNemar’s tests with Yate’s corrections. No differences were found between Helping and Re-enactment of Intended Acts, the easiest two tasks in all three groups. The next comparison indicated that Communication: Point task to be significantly more difficult in the CdLS (*p* = 0.04) and RTS (*p* = 0.04) groups, and Cooperation: Tubes in the FXS group (*p* = 0.02), than the Re-enactment of Intended Acts task. No further significant differences were found between any other task pairs. Figure 3 summarises these findings. These findings provide some evidence of structure in the performance of CdLS, FXS and RTS samples, but not enough differences to warrant testing whether the order of task difficulty formed a reliable alternative scale within any syndrome.

## Discussion

This is the first study to characterise the development of a broad range of early social cognitive abilities in neurogenetic syndromes that have been shown to have atypical profiles of autistic traits [16–18] using a novel technique utilising a normatively scaled battery of robust and established behavioural tasks [27]. Findings indicate that individuals with CdLS, FXS and RTS demonstrated a pattern of delay and difference in the development of early social cognitive abilities relative to that observed in TD or AUT comparison groups.

The first aim was to investigate whether individuals with CdLS, FXS and RTS develop early social cognitive skills at a rate that is advanced, similar or delayed relative to TD infants and AUT children. The development of intentionality abilities was delayed in children with CdLS, FXS and RTS relative to non-verbal mental age, suggesting that a mechanism other than overall general cognitive ability is disrupting social cognitive development. Individuals with CdLS showed a delay in passing these tasks that is comparable to AUT children and greater than the delay observed in individuals with FXS and RTS. However, the number of ESCogS tasks a participant passed was associated with higher non-verbal mental age in children with CdLS but not AUT children. Differences in intentionality between CdLS, FXS and AUT children, groups associated with social atypicalities, may underpin subtle differences observed between their behavioural and social phenotypes [8, 19, 29, 44].

The FXS and RTS groups showed a similar level of delay in social cognitive abilities, despite contrasting profiles of social and communication autistic characteristics. Whilst heightened sociability reported in individuals with RTS [45] may appear advantageous, individuals with RTS have also been described as ‘over friendly’ [44]. Compromised social cognitive ability may be associated with the ability to make judgements of another person’s trustworthiness [46] and when combined with high social motivation [45] may underpin why many individuals with RTS have been anecdotally reported by caregivers to be at a greater risk of exploitation by others. The results highlight the importance of detailed assessment of the profiles of cognitive and behavioural difference in individuals with RTS.

The second aim was to investigate whether participants in each syndrome group develop social cognitive abilities in the same order as that observed in TD and AUT children and/or one another or in a different order. Understanding differences in the developmental sequence can inform hypotheses on why early social cognition may be delayed in these groups [30]. Guttman scaling analyses provided no evidence that the syndrome groups developed these abilities in the same cumulative sequence previously observed in TD infants and AUT children [27]. Pairwise comparisons between tasks of increasing difficulty within each syndrome group provided some evidence that this was not due to high measurement error or variability within groups, but instead that the groups showed a distinctly different pattern. The first two easiest tasks (i.e., Helping and Re-enactment of Intended Acts) were significantly easier than the final four tasks (two Gestures tasks and two Cooperation tasks) in CdLS, FXS and RTS. Whereas in TD and AUT the Communication: Point task is no different in difficulty to Re-enactment of Intended Acts, it was more difficult for all three of the syndrome groups and appeared to be a specific atypicality.

One possibility is that all three syndrome groups have a specific difficulty with understanding the communicative function of pointing, although behavioural and clinical observations in these syndromes indicate broader profiles of social difference [29, 45]. The break in performance may reflect two sets of abilities that emerge from two distinct developmental streams hypothesised to lead to shared intentionality. These are: (1) a basic ability to understand other’s intentions and (2) a “species unique motivation” to share and represent others’ psychological states and to direct another’s attention to shared objects of interest [33]. Genetically determined neurobiological differences between CdLS, FXS and RTS may lead to disruption of later developing intentionality abilities assessed by the ESCogS.

Impairments in joint attention, a social referencing skill [47] that is a core precursor to social cognition and social interaction skills and behaviours [48, 49], may also contribute to the break in performance. Whilst participants can pass the two easiest ESCogS tasks by making inferences based on the examiner’s actions, the next four tasks require participants to either respond to the examiner’s use of joint attention, by indicating which box a toy is hidden in (both *Gestures* tasks) or initiate joint attention by directing the examiner’s attention so that they can fulfil their role in completing a joint goal (both Cooperation tasks). These findings correspond with previous findings indicating that infants with FXS initiated joint attention less frequently than TD infants and infant siblings of autistic children [50, 51] and children with FXS have demonstrated similar difficulties in initiating joint attention as children with non-syndromic ASC [52].

Intuitively, one may suppose that joint attention may be impaired by the extreme gaze aversion observed in those with FXS [53]. Individuals with CdLS and RTS also show differences in the frequency and quality of eye contact [29, 54], which may lead to compromised joint attention. However, infants with FXS are reported to use eye gaze during social initiations and responses and difficulties are more characterized by gesture impairments within these contexts (see [55] for a review). Thus, early gesture impairments are hypothesized to disrupt the later development of joint attention skills as well as social cognition skills, whereas gaze avoidance may not emerge until later childhood [55]. In contrast, those with CdLS have been reported to have intact gesture use [17, 56], but may be indicative of different underlying developmental causes of joint attention between neurogenetic syndromes.

Whilst the AUT group showed the same developmental sequence as TD children, it is notable that the pass rate (also reported in Table 2) for the three tasks on last two steps was “numerically lower” than the tasks on the first two steps, of which the authors suggested this may be a consequence of reduced eye contact and gaze following [27]. Males with non-syndromic ASC and males with fragile X show similar levels of eye contact [57] and AUT individuals show greater impairments in *responding* to others joint attention [52]. Yet the AUT children did not show the same level of difficulty in the Gestures: Point tasks [27] as the FXS group. This further supports the above argument that factors other than eye contact and gaze following may be sufficient for later developing intentionality skills, in this instance interpreting another’s communicative pointing gesture.

The heterogeneity in the patterns of pass and fails in individuals across all syndromes in the ESCogS (see Additional file 3) may be driven by the genetic heterogeneity within syndromes. CdLS [58, 59] and RTS [60] can be caused by variants of several different genes that are associated with differences in behavioural phenotypes [61, 62]. In FXS the number of CGG repeats correlates with the number of theory-of-mind tasks passed [23] and some individuals with FXS show genetic mosaicism, in which the number of cells affected by transcriptional silencing by the production of FMRP varies [63]. Mosaicism has also been found in individuals with CdLS with the NIPBL and more rarely the SMC3, RAD21 and SMC1A variants although no association between these mosaicism and clinical phenotype has been established yet [10].

Due to the rarity of the syndromes, the sample sizes were relatively small. Chronological age to the CdLS and FXS groups was not comparable to the RTS group. However, the focus is upon the *sequence* of the emergence of social cognitive abilities relative to individual’s ability rather than chronological age. To investigate the *developmental sequence* of early social cognitive abilities scale *within* syndromes, scaling analysis only requires that a cohort includes a wide range of ages and abilities that span the ages that TD children passed tasks [27, 64, 65]. Based on these aims, and due to the rarity of these syndromes, retaining the sample size was considered sufficient to conduct the study. No correlation was found between participant’s chronological age and the number of tasks they passed in any of the clinical groups, indicating that differences between groups in chronological age would not have a substantial influence on findings.

Whilst the syndrome groups were comparable on important socio-economic factors such as household income and main carers level of education, we did not collect data on race/ethnicity. Thus, we cannot comment on whether these factors may have influenced delay or difference in social cognitive abilities.

The field lacks cognitive assessments that have been normed and validated within populations with neurodevelopmental conditions and/or ID. Our approach was based on recent research at study design demonstrating good convergent validity between that Mullen Scales of Early Learning and the preschool form of the Differential Ability Scales [66, 67; the US normed version of the early year’s form of the BAS) on both verbal and non-verbal subscales in a sample of young autistic children and those with non-spectrum conditions with intellectual disability [68]. However, some participants in the current study performed at ceiling/floor effects on expressive language subscales of the Early Years BAS3 and School Age BAS3 respectively, indicating distinct strengths and weaknesses in language abilities in these groups [29]. Non-verbal mental age was used to characterise participant’s ability as this was available for most participant’s and thus best characterised the groups. Conclusions on the trajectory of social cognitive skills can only be made in relation to individual’s non-verbal mental age and not their verbal or overall broad level of ability. Whilst verbal ability is associated with a range of theory-of-mind tasks [69], the relationship between verbal ability and social cognition is not clear cut. For example, individuals with Down syndrome have relatively intact theory-of-mind abilities [70] but impaired receptive and expressive language deficits relative to their overall ability [71]. Research is needed to identify the relationship of both verbal and non-verbal ability with early social cognitive development per syndrome group.

We have provided further evidence of proof of concept that the utility of the ESCogS has been demonstrated through its identification of both delay and difference between these syndrome groups and AUT and TD comparisons. However, the lack of differences between the neurogenetic syndrome groups may indicate a lack of sensitivity in the ESCogS in detecting subtle but important differences in social cognitive development, a common challenge for many cognitive and behavioural measures applied to intellectual disability populations [72]. Nevertheless, the ESCogS has made a significant contribution by being the first and only developmental scale appropriate for children with an intellectual disability and limited language skills, utilising a range of observable assessments that do not have high language or cognitive demands.

## Conclusions

We presented the first study of early social cognitive development in children with CdLS, FXS and RTS and compared between syndromes, and TD and AUT comparison groups. Findings indicate differences in an etiological mechanism that may influence the different profiles of autistic traits observed between these groups and non-syndromic ASC. As well as evidence of a delay in these syndrome groups, the data raise the possibility of a discontinuity in the early social cognitive abilities of individuals with FXS, CdLS and RTS that differs from the pattern observed in TD or AUT. Findings have highlighted factors that may lead to disruption in early social cognitive development within these syndromes that may differ from non-syndromic AUT children. To distinguish between alternative interpretations of findings will require larger samples that confer the power to detect differences between individual tasks and to use scaling analysis to test for atypical but consistent patterns of performance within syndromes. Further work should seek to delineate the factors, such as the development of joint attention, eye gaze and gesture, that may disrupt early social cognition within each syndrome.

## Supplementary Information

**Additional file 1**. Social cognition in neurogenetic syndromes. Description of counterbalanced orders for Early Social Cognition Scale tasks

**Additional file 2**. ESCogS tasks. Description of the ability assessed, passing criteria and control trials for each task in the ESCogS

**Additional file 3**. Social cognition in neurogenetic syndromes. Pattern of pass and fails for each Early Social Cognition Scale task per participant in each syndrome group for the original scale

## Data Availability

The datasets used and/or analysed during the current study are available from the corresponding author on reasonable request.

## Abbreviations

ADOS-2: Autism Diagnostic Observation Schedule Second Edition
AUT: Autistic
BAS3: British Ability Scales Third Edition
CdLS: Cornelia de Lange syndrome
ESCogS: Early Social Cognition Scale
FXS: Fragile X syndrome
IoC: Index of conistency
Rep: Coefficient of reproducibility
RTS: Rubinstein–Taybi syndrome
TD: Typically developing
Vineland-II: Vineland Adaptive Behavior Scales Second Edition
