# Supplementary material for: The development of early social cognitive skills in neurogenetic syndromes associated with autism: Cornelia de Lange, fragile X and Rubinstein–Taybi syndromes
Source: Orphanet J Rare Dis. 2021 Nov 22;16:488. doi: 10.1186/s13023-021-02117-4 (PMC8607585; doi:10.1186/s13023-021-02117-4)
Supplement: Supplementary file 1 — Additional file 1. Social cognition in neurogenetic syndromes. Description of counterbalanced orders for Early Social Cognition Scale tasks [file 13023_2021_2117_MOESM1_ESM.docx]

**Description of counterbalanced orders for Early Social Cognition Scale tasks**

Counterbalanced orders (outlined in Table 1) were designed to ensure tasks were not in the same ordinal position and do not follow the same task more than twice. Tasks were split into two halves; the first half consisted of the three easiest tasks (Helping, Re-enactment of Intended Acts and Communication: Point) and was administered first to prevent participants from becoming disengaged or frustrated due to tasks being too difficult. The second half consisted of the two more difficult tasks (Cooperation: Tubes and Cooperation: Trampoline). The Helping experimental and control conditions were split between the two halves. The control was always administered in the first half to prevent the experimental condition priming helping in the control condition. Although Communication: Gaze is typically considered the same difficulty as Cooperation: Tubes, it was placed in the first half because it could only be administered at the same time as Gestures: Point, which is easier.

Table 1

| *Counterbalanced orders of tasks in the Early Social Cognition Scale* | | | | |  |
| --- | --- | --- | --- | --- | --- |
| Order 1 | Order 2 | Order 3 | Order 4 | Order 5 | Order 6 |
| *Re-enactment of Intended acts* | *Helping (control)* | *Helping (control)* | *Re-enactment of Intended acts* | *Gestures (Point & Gaze)* | *Gestures (Point & Gaze)* |
| *Helping (control)* | *Re-enactment of Intended acts* | *Gestures (Point & Gaze)* | *Gestures (Point & Gaze)* | *Re-enactment of Intended acts* | *Helping (control)* |
| *Gestures (Point & Gaze)* | *Gestures (Point & Gaze)* | *Re-enactment of Intended acts* | *Helping (control)* | *Helping (control)* | *Re-enactment of Intended acts* |
| *Cooperation –Tubes* | *Cooperation - Trampoline* | *Helping (experimental)* | *Cooperation –Tubes* | *Cooperation - Trampoline* | *Helping (experimental)* |
| *Cooperation - Trampoline* | *Cooperation –Tubes* | *Cooperation - Trampoline* | *Helping (experimental)* | *Helping (experimental)* | *Cooperation –Tubes* |
| *Helping (experimental)* | *Helping (experimental)* | *Cooperation –Tubes* | *Cooperation - Trampoline* | *Cooperation –Tubes* | *Cooperation - Trampoline* |
