# Supplementary material for: The development of early social cognitive skills in neurogenetic syndromes associated with autism: Cornelia de Lange, fragile X and Rubinstein–Taybi syndromes
Source: Orphanet J Rare Dis. 2021 Nov 22;16:488. doi: 10.1186/s13023-021-02117-4 (PMC8607585; doi:10.1186/s13023-021-02117-4)
Supplement: Supplementary file 2 — Additional file 2. ESCogS tasks. Description of the ability assessed, passing criteria and control trials for each task in the ESCogS [file 13023_2021_2117_MOESM2_ESM.docx]

| Descriptions of the ability assessed, passing criteria and control trials for each task in the ESCogS (Ellis et al., 2020) | | | |  | |
| --- | --- | --- | --- | --- | --- |
| Task | Ability assessed | Passing criteria | Corresponding control trial procedure | |  |
| *Helping* | Whether the participant understands another person’s basic intention and unachieved goals and show motivation to help that person achieve that goal | The infant picks up an item (either a pen or a polystyrene cone) that the experimenter reaches towards, which is close to the participant but out of reach from the experimenter and gives it to the experimenter, without any explicit prompts, in at least one out of two experimental trials. | Control trials follow the same procedure for each corresponding experimental trial, except the examiner does not reach for the item. | |  |
| *Re-enactment of Intended Acts* | Whether the participant understands another person's intentions by interpreting that person's goal-oriented, but unsuccessful action | The infant must carry out the examiner’s intended act, rather than imitating the examiner’s failed actions, in two out of three trials. |  | |  |
| *Gestures – Point* | Whether the participant understands that a communicative pointing gesture from experimenter two that indicates where experimenter one has hidden an object is intentionally directed towards the participant and is relevant to the current context. | The infant must choose the correct box with the toy hidden inside in both experimental trials, in which the second examiner indicates the location of the toy with a communicative and intentional pointing gesture. | Control trials follow the same procedure as experimental trials, except experimenter two does not indicate where the toy is hidden and instead experimenter one directs a non-intentionally, non-communicative distracted point towards where the object is hidden. | |  |
| *Gestures – Gaze* | Whether the participant understands that a communicative gaze gesture from experimenter two that indicates where experimenter one has hidden an object is intentionally directed towards the participant and is relevant to the current context. | The infant must choose the correct box with the toy hidden inside in both experimental trials, in which the second examiner indicates the location of the toy with a communicative and intentional gaze gesture. | Control trials follow the same procedure as experimental trials, except experimenter two does not indicate where the toy is hidden and instead experimenter one looks towards where the object is hidden in a non-intentional, non-communicative way. | |  |
| *Cooperation – Tubes* | Whether an infant can form a shared intentionality and cooperate with another person to achieve a joint goal in a problem-solving game to retrieve an object from inside a tube with handles, in which both partners must pull a handle from one side each to open the tubes. | The infant must: 1) show sufficient coordination to open the tubes with the examiner across all four trials and 2) show at least one attempt to reengage the examiner to complete the task during two interruption periods in which the examiner stops performing their role. |  | |  |
| *Cooperation – Trampoline* | Whether an infant can form a shared intentionality and cooperate with another person to achieve a joint goal in a social game, in which both partners must hold onto one side of a trampoline and bounce a block together. | The infant must: 1) be sufficiently engaged and successfully bounce the block on the trampoline with the examiner across all four trials and 2) show at least one attempt to reengage the examiner to complete the task during two interruption periods in which the examiner stops performing their role. |  | |  |
