# Supplementary material for: The development of early social cognitive skills in neurogenetic syndromes associated with autism: Cornelia de Lange, fragile X and Rubinstein–Taybi syndromes
Source: Orphanet J Rare Dis. 2021 Nov 22;16:488. doi: 10.1186/s13023-021-02117-4 (PMC8607585; doi:10.1186/s13023-021-02117-4)
Supplement: Supplementary file 3 — Additional file 3. Social cognition in neurogenetic syndromes. Pattern of pass and fails for each Early Social Cognition Scale task per participant in each syndrome group for the original scale [file 13023_2021_2117_MOESM3_ESM.docx]

# Pattern of pass and fails for each *Early Social Cognition Scale* task per participant in each syndrome group for the original scale.

|  | | | | |
| --- | --- | --- | --- | --- |
| Syndrome | Helping | REI or Point | Gaze or Tubes | Trampoline |
| CdLS | 0 | 0 | 0 | 0 |
|  | 1 | 1 | 0 | 0 |
|  | 1 | 1 | 0 | 0 |
|  | 1 | 1 | 0 | 0 |
|  | 1 | 1 | 1 | 1 |
|  | 1 | 0 | 0 | 0 |
|  | 1 | 1 | 1 | 0 |
|  | 1 | 1 | 0 | 0 |
|  | 0 | 0 | 0 | 0 |
|  | 0 | 0 | 1 | 0 |
|  | 1 | 1 | 0 | 0 |
|  | 0 | 1 | 0 | 0 |
|  | 0 | 1 | 0 | 0 |
|  | 1 | 1 | 0 | 0 |
|  | 1 | 1 | 0 | 0 |
|  | 1 | 1 | 1 | 1 |
|  | 1 | 0 | 0 | 0 |
|  | 1 | 0 | 0 | 0 |
|  | 0 | 1 | 0 | 0 |
|  | 0 | 1 | 0 | 0 |
|  | 0 | 1 | 0 | 0 |
|  | 1 | 1 | 1 | 0 |
| FXS | 0 | 0 | 1 | 0 |
|  | 1 | 1 | 1 | 0 |
|  | 0 | 0 | 1 | 0 |
|  | 1 | 0 | 1 | 0 |
|  | 1 | 1 | 0 | 0 |
|  | 1 | 1 | 0 | 0 |
|  | 1 | 1 | 0 | 0 |
|  | 1 | 1 | 1 | 0 |
|  | 1 | 1 | 0 | 0 |
|  | 0 | 1 | 0 | 0 |
|  | 1 | 1 | 0 | 0 |
|  | 1 | 1 | 1 | 0 |
|  | 1 | 1 | 1 | 0 |
|  | 1 | 1 | 0 | 1 |
|  | 1 | 1 | 0 | 0 |
|  | 1 | 1 | 1 | 1 |
|  | 1 | 1 | 0 | 0 |
|  | 1 | 1 | 1 | 0 |
|  | 1 | 1 | 1 | 1 |
| RTS | 1 | 1 | 0 | 1 |
|  | 0 | 1 | 0 | 0 |
|  | 1 | 1 | 0 | 0 |
|  | 1 | 1 | 1 | 1 |
|  | 1 | 1 | 1 | 1 |
|  | 1 | 1 | 1 | 1 |
|  | 1 | 1 | 1 | 1 |
|  | 1 | 1 | 0 | 0 |
|  | 1 | 0 | 0 | 0 |
|  | 1 | 1 | 1 | 0 |
|  | 1 | 1 | 1 | 1 |
|  | 1 | 1 | 0 | 1 |
|  | 0 | 1 | 0 | 0 |
|  | 1 | 1 | 0 | 0 |
|  | 1 | 1 | 0 | 0 |
|  | 1 | 1 | 0 | 0 |
|  | 1 | 1 | 1 | 0 |
|  | 1 | 0 | 0 | 0 |
